# Supplementary material for: Discovery and application of insertion-deletion (INDEL) polymorphisms for QTL mapping of early life-history traits in Atlantic salmon
Source: BMC Genomics. 2010 Mar 8;11:156. doi: 10.1186/1471-2164-11-156 (PMC2838853; doi:10.1186/1471-2164-11-156)
Supplement: Additional file 2 — Information on developed 76 locus single-run INDEL panel in Atlantic salmon. Information on fluorescence labeling, primer concentrations, PCR pooling and links to alignments, INDEL motifs and GENESCAN (Burge and Karlin 1997) predictions of genes/exons are available in html format. [file 1471-2164-11-156-S2.ZIP › Additionalfile2/table8570.html]

Contig Alignment

```
Base	A B C D E F G H I J K L M N O P Q R S T U   
1	A A . A A A A A A A A A . A A . . . . . .    
2	A A A A A A A A A A A A . A A A A . . . .    
3	A A A A A A A A A A A A . A A A A . . . .    
4	C C C C C C C C C C C C C C C C C . . . .    
5	G G G G G G G G G G G G G G G G G . . . .    
6	T T T T T T T T T T T T T T T T T . . . .    
7	A A A A A A A A A A A A A A A A A . . A A    
8	G G G G G G G G G G G G G G G G G . . G G    
9	A A A A A A A A A A A A A A A A A . . A A    
10	A A A A A A A A A A A A A A A A A . . A A    
11	A A A A A A A A A A A A A A A A A . . A A    
12	C C C C C C C C C C C C C C C C C . . C C    
13	A A A A A A A A A A A A A A A A A . . A A    
14	A A A A A A A A A A A A A A A A A . . A A    
15	T T T T T T T T T T T T T T T T T . . T T    
16	A A A A A A A A A A A A A A A A A . . A A    
17	T T T T T T T T T T T T T T T T T . . T T    
18	T T T T T T T T T T T T T T T T T . . T T    
19	T T T T T T T T T T T T T T T T T . . T T    
20	A A A A A A A A A A A A A A A A A . . A A    
21	T T T T T T T T T T T T T T T T T . . T T    
22	T T T T T T T T T T T T T T T T T . . T T    
23	C C C C C C C C C C C C C C C C C . . C C    
24	T T T T T T T T T T T T T T T T T . . T T    
25	T T T T T T T T T T T T T T T T T . . T T    
26	C C C C C C C C C C C C C C C C C . . C C    
27	A A A A A A A A A A A A A A A A A . . A A    
28	T T T T T T T T T T T T T T T T T . . T T    
29	A A A A A A A A A A A A A A A A A . . A A    
30	G G G G G G G G G G G G G G G G G . . G G    
31	A A A A A A A A A A A A A A A A A . . A A    
32	C C C C C C C C C C C C C C C C C . . C C    
33	A A A A A A A A A A A A A A A A A . . A A    
34	T T T T T T T T T T T T T T T T T . . T T    
35	A A A A A A A A A A A A A A A A A . . A A    
36	C C C C C C C C C C C C C C C C C . . C C    
37	A A A A A A A A A A A A A A A A A . . A A    
38	A A A A A A A A A A A A A A A A A . . A A    
39	A A A A A A A A A A A A A A A A A . . A A    
40	A A A A A A A A A A A A A A A A A . . A A    
41	G G G G G G G G G G G G G G G G G . . G G    
42	C C C C C C C C C C C C C C C C C . . C C    
43	A A A A A A A A A A A A A A A A A . . A A    
44	G G G G G G G G G G G G G G G G G . . G G    
45	G G G G G G G G G G G G G G G G G . . G G    
46	T T T T T T T T T T T T T T T T T . . T T    
47	G G G G G G G G G G G G G G G G G . . G G    
48	A A A A A A A A A A A A A A A A A . . A A    
49	A A A A A A A A A A A A A A A A A . . A A    
50	A A A A A A A A A A A A A A A A A . . A A    
51	G G G G G G G G G G G G G G G G G . . G G    
52	A A A A A A A A A A A A A A A A A . . A A    
53	C C C C C C C C C C C C C C C C C . . C C    
54	T T T T T T T T T T T T T T T T T . . T T    
55	T T T T T T T T T T T T T T T T T . . T T    
56	T T T T T T T T T T T T T T T T T . . T T    
57	A A A A A A A A A A A A A A A A A . . A A    
58	C C C C C C C C C C C C C C C C C C . C C    
59	A A A A A A A A A A A A A A A A A A . A A    
60	G G G G G G G G G G G G G G G G G G . G G    
61	C C C C C C C C C C C C C C C C C C . C C    
62	G G G G G G G G G G G G G G G G G G . G G    
63	C C C C C C C C C C C C C C C C C C . C C    
64	T T T T T T T T T T T T T T T T T T . T T    
65	A A A A A A A A A A A A A A A A A A . A A    
66	T T T T T T T T T T T T T T T T T T . T T    
67	T T T T T T T T T T T T T T T T T T . T T    
68	T T T T T T T T T T T T T T T T T T . T T    
69	G G G G G G G G G G G G G G G G G G . G G    
70	G G G G G G G G G G G G G G G G G G . G G    
71	A A A A A A A A A A A A A A A A A A . A A    
72	T T T T T T T T T T T T T T T T T T . T T    
73	A A A A A A A A A A A A A A A A A A . A A    
74	A A A A A A A A A A A A A A A A A A . A A    
75	T T T T T T T T T T T T T T T T T T . T T    
76	A A A A A A A A A A A A A A A A A A . A A    
77	T T T T T T T T T T T T T T T T T T . T T    
78	G G G G G G G G G G G G G G G G G G . G G    
79	A A A A A A A A A A A A A A A A A A . A A    
80	A A A A A A A A A A A A A A A A A A . A A    
81	C C C C C C C C C C C C C C C C C C . C C    
82	A A A A A A A A A A A A A A A A A A . A A    
83	A A A A A A A A A A A A A A A A A A . A A    
84	G G G G G G G G G G G G G G G G G G . G G    
85	A A A A A A A A A A A A A A A A A A . A A    
86	A A A A A A A A A A A A A A A A A A . A A    
87	G G G G G G G G G G G G G G G G G G . G G    
88	A A A A A A A A A A A A A A A A A A . A A    
89	G G G G G G G G G G G G G G G G G G . G G    
90	A A A A A A A A A A A A A A A A A A . A A    
91	A A A A A A A A A A A A A A A A A A . A A    
92	C C C C C C C C C C C C C C C C C C . C C    
93	A A A A A A A A A A A A A A A A A A . A A    
94	C C C C C C C C C C C C C C C C C C . C C    
95	A A A A A A A A A A A A A A A A A A . A A    
96	A A A A A A A A A A A A A A A A A A . A A    
97	C C C C C C C C C C C C C C C C C C . C C    
98	A A A A A A A A A A A A A A A A A A . A A    
99	G G G G G G G G G G G G G G G G G G . G G    
100	T T T T T T T T T T T T T T T T T T . T T    
101	A A A A A A A A A A A A A A A A A A . A A    
102	A A A A A A A A A A A A A A A A A A . A A    
103	A A A A A A A A A A A A A A A A A A . A A    
104	A A A A A A A A A A A A A A A A A A . A A    
105	G G G G G G G G G G G G G G G G G G . G G    
106	C C C C C C C C C C C C C C C C C C . C C    
107	A A A A A A A A A A A A A A A A A A . A A    
108	G G G G G G G G G G G G G G G G G G . G G    
109	C C C C C C C C C C C C C C C C C C . C C    
110	A A A A A A A A A A A A A A A A A A . A A    
111	A A A A A A A A A A A A A A A A A A . A A    
112	T T T T T T T T T T T T T T T T T T . T T    
113	A A A A A A A A A A A A A A A A A A . A A    
114	T T T T T T T T T T T T T T T T T T . T T    
115	T T T T T T T T T T T T T T T T T T . T T    
116	G G G G G G G G G G G G G G G G G G . G G    
117	T T T T T T T T T T T T T T T T T T . T T    
118	A A A A A A A A A A A A A A A A A A . A A    
119	A A A A A A A A A A A A A A A A A A . A A    
120	A A A A A A A A A A A A A A A A A A . A A    
121	A A A A A A A A A A A A A A A A A A . A A    
122	G G G G G G G G G G G G G G G G G G . G G    
123	A A A A A A A A A A A A A A A A A A . A A    
124	G G G G G G G G G G G G G G G G G G . G G    
125	G G G G G G G G G G G G G G G G G G . G G    
126	G G G G G G G G G G G G G G G G G G . G G    
127	A A A A A A A A A A A A A A A A A A . A A    
128	C C C C C C C C C C C C C C C C C C . C C    
129	A A A A A A A A A A A A A A A A A A . A A    
130	G G G G G G G G G G G G G G G G G G . G G    
131	A A A A A A A A A A A A A A A A A A . A A    
132	G G G G G G G G G G G G G G G G G G . G G    
133	A A A A A A A A A A A A A A A A A A . A A    
134	G G G G G G G G G G G G G G G G G G . G G    
135	A A A A A A A A A A A A A A A A A A . A A    
136	C C C C C C C C C C C C C C C C C C . C C    
137	A A A A A A A A A A A A A A A A A A . A A    
138	C C C C C C C C C C C C C C C C C C . C C    
139	C C C C C C C C C C C C C C C C C C . C C    
140	A A A A A A A A A A A A A A A A A A . A A    
141	T T T T T T T T T T T T T T T T T T . T T    
142	T T T T T T T T T T T T T T T T T T . T T    
143	A A A A A A A A A A A A A A A A A A . A A    
144	G G G G G G G G G G G G G G G G G G . G G    
145	A A A A A A A A A A A A A A A A A A . A A    
146	T T T T T T T T T T T T T T T T T T . T T    
147	G G G G G G G G G G G G G G G G G G . G G    
148	T T T T T T T T T T T T T T T T T T . T T    
149	C C C C C C C C C C C C C C C C C C . C C    
150	A A A A A A A A A A A A A A A A A A . A A    
151	C C C C C C C C C C C C C C C C C C . C C    
152	T T T T T T T T T T T T T T T T T T . T T    
153	G G G G G G G G G G G G G G G G G G . G G    
154	T T T T T T T T T T T T T T T T T T . T T    
155	G G G G G G G G G G G G G G G G G G . G G    
156	T T T T T T T T T T T T T T T T T T . T T    
157	A A A A A A A A A A A A A A A A A A . A A    
158	T T T T T T T T T T T T T T T T T T . T T    
159	C C C C C C C C C C C C C C C C C C . C C    
160	C C C C C C C C C C C C C C C C C C . C C    
161	A A A A A A A A A A A A A A A A A A . A A    
162	G G G G G G G G G G G G G G G G G G . G G    
163	G G G G G G G G G G G G G G G G G G . G G    
164	C C C C C C C C C C C C C C C C C C . C C    
165	C C C C C C C C C C C C C C C C C C . C C    
166	A A A A A A A A A A A A A A A A A A . A A    
167	G G G G G G G G G G G G G G G G G G . G G    
168	A A A A A A A A A A A A A A A A A A . A A    
169	G G G G G G G G G G G G G G G G G G . G G    
170	G G G G G G G G G G G G G G G G G G . G G    
171	A A A A A A A A A A A A A A A A A A . A A    
172	G G G G G G G G G G G G G G G G G G . G G    
173	C C C C C C C C C C C C C C C C C C . C C    
174	T T T T T T T T T T T T T T T T T T . T T    
175	T T T T T T T T T T T T T T T T T T . T T    
176	G G G G G G G G G G G G G G G G G G . G G    
177	A A A A A A A A A A A A A A A A A A . A A    
178	C C C C C C C C C C C C C C C C C C . C C    
179	G G G G G G G A A G G G G G G G G G . G G    
180	C C C C C C C C C C C C C C C C C C . C C    
181	T T T T T T T T T T T T T T T T T T . T T    
182	T T T T T T T T T T T T T T T T T T . T T    
183	G G G G G G G G G G G G G G G G G G . G G    
184	G G G G G G G G G G G G G G G G G G . G G    
185	G G G G G G G G G G G G G G G G G G . G G    
186	T T T T T T T T T T T T T T T T T T . T T    
187	C C C C C C C C C C C C C C C C C C . C C    
188	A A A A A A A A A A A A A A A A A A . A A    
189	T T T T T T T T T T T T T T T T T T . T T    
190	C C C C C C C C C C C C C C C C C C . C C    
191	A A A A A A A A A A A A A A A A A A . A A    
192	G G G G G G G G G G G G G G G G G G . G G    
193	A A A A A A A A A A A A A A A A A A . A A    
194	G G G G G G G G G G G G G G G G G G . G G    
195	G G G G G G G G G G G G G G G G G G . G G    
196	A A A A A A A A A A A A A A A A A A . A A    
197	A A A A A A A A A A A A A A A A A A . A A    
198	G G G G G G G G G G G G G G G G G G . G G    
199	G G G G G G G G G G G G G G G G G G . G G    
200	A A A A A A A A A A A A A A A A A A . A A    
201	C C C C C C C C C C C C C C C C C C . C C    
202	T T T T T T T T T T T T T T T T T T . T T    
203	A A A A A A A A A A A A A A A A A A . A A    
204	G G G G G G G G G G G G G G G G G G . G G    
205	G G G G G G G G G G G G G G G G G G . G G    
206	G G G G G G G G G G G G G G G G G G . G G    
207	A A A A A A A A A A A A A A A A A A . A A    
208	G G G G G G G G G G G G G G G G G G . G G    
209	C C C C C C C C C C C C C C C C C C . C C    
210	A A A A A A A A A A A A A A A A A A . A A    
211	A A A A A A A A A A A A A A A A A A . A A    
212	C C C C C C C C C C C C C C C C C C . C C    
213	C C C C C C C C C C C C C C C C C C . C C    
214	C C C C C C C C C C C C C C C C C C . C C    
215	G G G G G G G G G G G G G G G G G G . G G    
216	G G G G G G G G G G G G G G G G G G . G G    
217	T T T T T T T T T T T T T T T T T T . T T    
218	T T T T T T T T T T T T T T T T T T . T T    
219	G G G G G G G G G G G G G G G G G G . G G    
220	G G G G G G G G G G G G G G G G G G . G G    
221	T T T T T T T T T T T T T T T T T T . T T    
222	C C C C C C C C C C C C C C C C C C . C C    
223	A A A A A A A A A A A A A A A A A A . A A    
224	G G G G G G G G G G G G G G G G G G . G G    
225	T T T T T T T T T T T T T T T T T T . T T    
226	C C C C C C C C C C C C C C C C C C . C C    
227	C C C C C C C C C C C C C C C C C C . C C    
228	T T T T T T T T T T T T T T T T T T . T T    
229	C C C C C C C C C C C C C C C C C C . C C    
230	A A A A A A A A A A A A A A A A A A . A A    
231	T T T T T T T T T T T T T T T T T T . T T    
232	C C C C C C C C C C C C C C C C C C . C C    
233	A A A A A A A A A A A A A A A A A A . A A    
234	T T T T T T T T T T T T T T T T T T . T T    
235	T T T T T T T T T T T T T T T T T T . T T    
236	C C C C C C C C C C C C C C C C C C . C C    
237	T T T T T T T T T T T T T T T T T T . T T    
238	G G G G G G G G G G G G G G G G G G . G G    
239	C C C C C C C C C C C C C C C C C C . C C    
240	A A A A A A A A A A A A A A A A A A . A A    
241	T T T T T T T T T T T T T T T T T T . T T    
242	C C C C C C C C C C C C C C C C C C . C C    
243	C C C C C C C C C C C C C C C C C C . C C    
244	A A A A A A A A A A A A A A A A A A . A A    
245	G G G G G G G G G G G G G G G G G G . G G    
246	C C C C C C C C C C C C C C C C C C . C C    
247	T T T T T T T T T T T T T T T T T T . T T    
248	G G G G G G G G G G G G G G G G G G . G G    
249	C C C C C C C C C C C C C C C C C C . C C    
250	T T T T T T T T T T T T T T T T T T . T T    
251	A A A A A A A A A A A A A A A A A A . A A    
252	C C C C C C C C C C C C C C C C C C . C C    
253	A A A A A A A A A A A A A A A A A A . A A    
254	A A A A A A A A A A A A A A A A A A . A A    
255	A A A A A A A A A A A A A A A A A A . A A    
256	C C C C C C C C C C C C C C C C C C . C C    
257	C C C C C C C C C C C C C C C C C C . C C    
258	A A A A A A A A A A A A A A A A A A . A A    
259	C C C C C C C C C C C C C C C C C C . C C    
260	G G G G G G G G G G G G G G G G G G . G G    
261	G G G G G G G G G G G G G G G G G G . G G    
262	A A A A A A A A A A A A A A A A A A . A A    
263	T T T T T T T T T T T T T T T T T T . T T    
264	G G G G G G G G G G G G G G G G G G . G G    
265	A A A A A A A A A A A A A A A A A A . A A    
266	C C C C C C C C C C C C C C C C C C . C C    
267	T T T T T T T T T T T T T T T T T T . T T    
268	G G G G G G G G G G G G G G G G G G . G G    
269	G G G G G G G G G G G G G G G G G G . G G    
270	C C C C C C C C C C C C C C C C C C . C C    
271	T T T T T T T T T T T T T T T T T T . T T    
272	T T T T T T T T T T T T T T T T T T T T T    
273	C C C C C C C C C C C C C C C C C C C C C    
274	A A A A A A A A A A A A A A A A A A A A A    
275	T T T T T T T T T T T T T T T T T T T T T    
276	C C C C C C C C C C C C C C C C C C C C C    
277	A A A A A A A A A A A A A A A A A A A A A    
278	A A A A A A A A A A A A A A A A A A A A A    
279	C C C C C C C C C C C C C C C C C C C C C    
280	G G G G G G G G G G G G G G G G G G G G G    
281	G G G G G G G G G G G G G G G G G G G G G    
282	T T T T T T T T T T T T T T T T T T T T T    
283	G G G G G G G G G G G G G G G G G G G G G    
284	T T T T T T T T T T T T T T T T T T T T T    
285	C C C C C C C C C C C C C C C C C C C C C    
286	T T T T T T T T T T T T T T T T T T T T T    
287	A A A A A A A A A A A A A A A A A A A A A    
288	T T T T T T T T T T T T T T T T T T T T T    
289	T T T T T T T T T T T T T T T T T T T T T    
290	C C C C C C C C C C C C C C C C C C C C C    
291	A A A A A A A A A A A A A A A A A A A A A    
292	G G G G G G G G G G G G G G G G G G G G G    
293	T T T T T T T T T T T T T T T T T T T T T    
294	C C C C C C C C C C C C C C C C C C C C C    
295	T T T T T T T T T T T T T T T T T T T T T    
296	T T T T T T T T T T T T T T T T T T T T T    
297	C C C C C C C C C C C C C C C C C C C C C    
298	A A A A A A A A A A A A A A A A A A A A A    
299	G G G G G G G G G G G G G G G G G G G G G    
300	T T T T T T T T T T T T T T T T T T T T T    
301	G G G G G G G G G G G G G G G G G G G G G    
302	C C C C C C C C C C C C C C C C C C C C C    
303	C C C C C C C C C C C C C C C C C C C C C    
304	T T T T T T T T T T T T T T T T T T T T T    
305	T T T T T T T T T T T T T T T T T T T T T    
306	C C C C C C C C C C C C C C C C C C C C C    
307	T T T T T T T T T T T T T T T T T T T T T    
308	C C C C C C C C C C C C C C C C C C C C C    
309	C C C C C C C C C C C C C C C C C C C C C    
310	A A A A A A A A A A A A A A A A A A A A A    
311	G G G G G G G G G G G G G G G G G G G G G    
312	G G G G G G G G G G G G G G G G G G G G G    
313	G G G G G G G G G G G G G G G G G G G G G    
314	C C C C C C C C C C C C C C C C C C C C C    
315	A A A A A A A A A A A A A A A A A A A A A    
316	G G G G G G G G G G G G G G G G G G G G G    
317	A A A A A A A A A A A A A A A A A A A A A    
318	A A A A A A A A A A A A A A A A A A A A A    
319	A A A A A A A A A A A A A A A A A A A A A    
320	C C C C C C C C C C C C C C C C C C C C C    
321	T T T T T T T T T T T T T T T T T T T T T    
322	A A A A A A A A A A A A A A A A A A A A A    
323	C C C C C C C C C C C C C C C C C C C C C    
324	A A A A A A A A A A A A A A A A A A A A A    
325	A A A A A A A A A A A A A A A A A A A A A    
326	C C C C C C C C C C C C C C C C C C C C C    
327	T T T T T T T T T T T T T T T T T T T T T    
328	T T T T T T T T T T T T T T T T T T T T T    
329	C C C C C C C C C C C C C C C C C C C C C    
330	A A A A A A A A A A A A A A A A A A A A A    
331	G G G G G G G G G G G G G G G G G G G G G    
332	G G G G G G G G G G G G G G G G G G G G G    
333	C C C C C C C C C C C C C C C C C C C C C    
334	T T T T T T T T T T T T T T T T T T T T T    
335	C C C C C C C C C C C C C C C C C C C C C    
336	A A A A A A A A A A A A A A A A A A A A A    
337	G G G G G G G G G G G G G G G G G G G G G    
338	G G G G G G G G G G G G G G G G G G G G G    
339	A A A A A A A A A A A A A A A A A A A A A    
340	A A A A A A A A A A A A A A A A A A A A A    
341	A A A A A A A A A A A A A A A A A A A A A    
342	C C C C C C C C C C C C C C C C C C C C C    
343	T T T T T T T T T T T T T T T T T T T T T    
344	T T T T T T T T T T T T T T T T T T T T T    
345	C C C C C C C C C C C C C C C C C C C C C    
346	A A A A A A A A A A A A A A A A A A A A A    
347	G G G G G G G G G G G G G G G G G G G G G    
348	C C C C C C C C C C C C C C C C C C C C C    
349	T T T T T T T T T T T T T T T T T T T T T    
350	T T T T T T T T T T T T T T T T T T T T T    
351	G G G G G G G G G G G G G G G G G G G G G    
352	C C C C C C C C C C C C C C C C C C C C C    
353	G G G G G G G G G G G G G G G G G G G G G    
354	A A A A A A A A A A A A A A A A A A A A A    
355	G G G G G G G G G G G G G G G G G G G G G    
356	G G G G G G G G G G G G G G G G G G G G G    
357	C C C C C C C C C C C C C C C C C C C C C    
358	G G G G G G G G G G G G G G G G G G G G G    
359	G G G G G G G G G G G G G G G G G G G G G    
360	G G G G G G G G G G G G G G G G G G G G G    
361	C C C C C C C C C C C C C C C C C C C C C    
362	C C C C C C C C C C C C C C C C C C C C C    
363	T T T T T T T T T T T T T T T T T T T T T    
364	T T T T T T T T T T T T T T T T T T T T T    
365	T T T T T T T T T T T T T T T T T T T T T    
366	C C C C C C C C C C C C C C C C C C C C C    
367	T T T T T T T T T T T T T T T T T T T T T    
368	T T T T T T T T T T T T T T T T T T T T T    
369	T T T T T T T T T T T T T T T T T T T T T    
370	A A A A A A A A A A A A A A A A A A A A A    
371	T T T T T T T T T T T T T T T T T T T T T    
372	C C C C C C C C C C C C C C C C C C C C C    
373	C C C C C C C C C C C C C C C C C C C C C    
374	C C C C C C C C C C C C C C C C C C C C C    
375	T T T T T T T T T T T T T T T T T T T T T    
376	G G G G G G G G G G G G G G G G G G G G G    
377	A A A A A A A A A A A A A A A A A A A A A    
378	C C C C C C C C C C C C C C C C C C C C C    
379	C C C C C C C C C C C C C C C C C C C C C    
380	A A A A A A A A A A A A A A A A A A A A A    
381	C C C C C C C C C C C C C C C C C C C C C    
382	A A A A A A A A A A A A A A A A A A A A A    
383	G G G G G G G G G G G G G G G G G G G G G    
384	A A A A A A A A A A A A A A A A A A A A A    
385	C C C C C C C C C C C C C C C C C C C C C    
386	A A A A A A A A A A A A A A A A A A A A A    
387	G G G G G G G G G G G G G G G G G G G G G    
388	A A A A A A A A A A A A A A A A A A A A A    
389	C C C C C C C C C C C C C C C C C C C C C    
390	C C C C C C C C C C C C C C C C C C C C C    
391	T T T T T T T T T T T T T T T T T T T T T    
392	C C C C C C C C C C C C C C C C C C C C C    
393	T T T T T T T T T T T T T T T T T T T T T    
394	T T T T T T T T T T T T T T T T T T T T T    
395	T T T T T T T T T T T T T T T T T T T T T    
396	C C C C C C C C C C C C C C C C C C C C C    
397	T T T T T T T T T T T T T T T T T T T T T    
398	C C C C C C C C C C C C C C C C C C C C C    
399	T T T T T T T T T T T T T T T T T T T T T    
400	C C C C C C C C C C C C C C C C C C C C C    
401	C C C C C C C C C C C C C C C C C C C C C    
402	T T T T T T T T T T T T T T T T T T T T T    
403	T T T T T T T T T T T T T T T T T T T T T    
404	C C C C C C C C C C C C C C C C C C C C C    
405	A A A A A A A A A A A A A A A A A A A A A    
406	A A A A A A A A A A A A A A A A A A A A A    
407	C C C C C C C C C C C C C C C C C C C C C    
408	C C C C C C C C C C C C C C C C C C C C C    
409	A A A A A A A A A A A A A A A A A A A A A    
410	G G G G G G G G G G G G G G G G G G G G G    
411	A A A A A A A A A A A A A A A A A A A A A    
412	A A A A A A A A A A A A A A A A A A A A A    
413	T T T T T T T T T T T T T T T T T T T T T    
414	C C C C C C C C C C C C C C C C C C C C C    
415	A A A A A A A A A A A A A A A A A A A A A    
416	C C C C C C C C C C C C C C C C C C C C C    
417	T T T T T T T T T T T T T T T T T T T T T    
418	T T T T T T T T T T T T T T T T T T T T T    
419	C C C C C C C C C C C C C C C C C C C C C    
420	A A A A A A A A A A A A A A A A A A A A A    
421	A A A A A A A A A A A A A A A A A A A A A    
422	A A A A A A A A A A A A A A A A A A A A A    
423	A A A A A A A A A A A A A A A A A A A A A    
424	C C C C C C C C C C C C C C C C C C C C C    
425	T T T T T T T T T T T T T T T T T T T T T    
426	T T T T T T T T T T T T T T T T T T T T T    
427	T T T T T T T T T T T T T T T T T T T T T    
428	T T T T T T T T T T T T T T T T T T T T T    
429	A A A A A A A A A A A A A A A A A A A A A    
430	C C C C C C C C C C C C C C C C C C C C C    
431	T T T T T T T T T T T T T T T T T T T T T    
432	C C C C C C C C C C C C C C C C C C C C C    
433	C C C C C C C C C C C C C C C C C C C C C    
434	G G G G G G G G G G G G G G G G G G G G G    
435	G G G G G G G G G G G G G G G G G G G G G    
436	G G G G G G G G G G G G G G G G G G G G G    
437	G G G G G G G G G G G G G G G G G G G G G    
438	C C C C C C C C C C C C C C C C C C C C C    
439	T T T T T T T T T T T T T T T T T T T T T    
440	T T T T T T T T T T T T T T T T T T T T T    
441	C C C C C C C C C C C C C C C C C C C C C    
442	T T T T T T T T T T T T T T T T T T T T T    
443	G G G G G G G G G G G G G G G G G G G G G    
444	G G G G G G G G G G G G G G G G G G G G G    
445	G G G G G G G G G G G G G G G G G G G G G    
446	G G G G G G G G G G G G G G G G G G G G G    
447	G G G G G G G G G G G G G G G G G G G G G    
448	T T T T T T T T T T T T T T T T T T T T T    
449	T T T T T T T T T T T T T T T T T T T T T    
450	C C C C C C C C C C C C C C C C C C C C C    
451	A A A A A A A A A A A A A A A A A A A A A    
452	G G G G G G G G G G G G G G G G G G G G G    
453	G G A G G G G G G A G G A A A A A A A G G  9	 transition  
454	A A A A A A A A A A A A A A A A A A A A A    
455	C C C C C C C C C C C C C C C C C C C C C    
456	C C C C C C C C C C C C C C C C C C C C C    
457	A A A A A A A A A A A A A A A A A A A A A    
458	C C C C C C C C C C C C C C C C C C C C C    
459	A A A A A A A A A A A A A A A A A A A A A    
460	G G G G G G G G G G G G G G G G G G G G G    
461	T T T T T T T T T T T T T T T T T T T T T    
462	G G G G G G G G G G G G G G G G G G G G G    
463	A A A A A A A A A A A A A A A A A A A A A    
464	G G G G G G G G G G G G G G G G G G G G G    
465	G G G G G G G G G G G G G G G G G G G G G    
466	T T T T T T T T T T T T T T T T T T T T T    
467	C C C C C C C C C C C C C C C C C C C C C    
468	T T T T T T T T T T T T T T T T T T T T T    
469	G G G G G G G G G G G G G G G G G G G G G    
470	G G G G G G G G G G G G G G G G G G G G G    
471	A A A A A A A A A A A A A A A A A A A A A    
472	C C C C C C C C C C C C C C C C C C C C C    
473	A A A A A A A A A A A A A A A A A A A A A    
474	G G G G G G G G G G G G G G G G G G G G G    
475	G G G G G G G G G G G G G G G G G G G G G    
476	C C C C C C C C C C C C C C C C C C C C C    
477	A A A A A A A A A A A A A A A A A A A A A    
478	G G G G G G G G G G G G G G G G G G G G G    
479	C C C C C C C C C C C C C C C C C C C C C    
480	C C C C C C C C C C C C C C C C C C C C C    
481	A A A A A A A A A A A A A A A A A A A A A    
482	G G G G G G G G G G G G G G G G G G G G G    
483	G G G G G G G G G G G G G G G G G G G G G    
484	A A A A A A A A A A A A A A A A A A A A A    
485	G G G G G G G G G G G G G G G G G G G G G    
486	G G G G G G G G G G G G G G G G G G G G G    
487	G G G G G G G G G G G G G G G G G G G G G    
488	C C C C C C C C C C C C C C C C C C C C C    
489	A A A A A A A A A A A A A A A A A A A A A    
490	A A A A A A A A A A A A A A A A A A A A A    
491	C C C C C C C C C C C C C C C C C C C C C    
492	T T T T T T T T T T T T T T T T T T T T T    
493	C C C C C C C C C C C C C C C C C C C C C    
494	T T T T T T T T T T T T T T T T T T T T T    
495	G G G G G G G G G G G G G G G G G G G G G    
496	A A A A A A A A A A A A A A A A A A A A A    
497	C C C C C C C C C C C C C C C C C C C C C    
498	C C C C C C C C C C C C C C C C C C C C C    
499	C C C C C C C C C C C C C C C C C C C C C    
500	C C C C C C C C C C C C C C C C C C C C C    
501	C C C C C C C C C C C C C C C C C C C C C    
502	T T T T T T T T T T T T T T T T T T T T T    
503	C C C C C C C C C C C C C C C C C C C C C    
504	G G G G G G G G G G G G G G G G G G G G G    
505	G G G G G G G G G G G G G G G G G G G G G    
506	C C C C C C C C C C C C C C C C C C C C C    
507	A A A A A A A A A A A A A A A A A A A A A    
508	T T T T T T T T T T T T T T T T T T T T T    
509	T T T T T T T T T T T T T T T T T T T T T    
510	A A A A A A A A A A A A A A A A A A A A A    
511	C C C C C C C C C C C C C C C C C C C C C    
512	G G G G G G G G G G G G G G G G G G G G G    
513	C C C C C C C C C C C C C C C C C C C C C    
514	C C C C C C C C C C C C C C C C C C C C C    
515	C C C C C C C C C C C C C C C C C C C C C    
516	G G G G G G G G G G G G G G G G G G G G G    
517	T T T T T T T T T T T T T T T T T T T T T    
518	A A A A A A A A A A A A A A A A A A A A A    
519	C C C C C C C C C C C C C C C C C C C C C    
520	A A A A A A A A A A A A A A A A A A A A A    
521	C C C C C C C C C C C C C C C C C C C C C    
522	T T T T T T T T T T T T T T T T T T T T T    
523	C C C C C C C C C C C C C C C C C C C C C    
524	G G G G G G G G G G G G G G G G G G G G G    
525	T T T T T T T T T T T T T T T T T T T T T    
526	C C C C C C C C C C C C C C C C C C C C C    
527	A A A A A A A A A A A A A A A A A A A A A    
528	G G G G G G G G G G G G G G G G G G G G G    
529	C C C C C C C C C C C C C C C C C C C C C    
530	T T T T T T T T T T T T T T T T T T T T T    
531	T T T T T T T T T T T T T T T T T T T T T    
532	T T T T T T T T T T T T T T T T T T T T T    
533	T T T T T T T T T T T T T T T T T T T T T    
534	A A A A A A A A A A A A A A A A A A A A A    
535	C C C C C C C C C C C C C C C C C C C C C    
536	A A A A A A A A A A A A A A A A A A A A A    
537	G G G G G G G G G G G G G G G G G G G G G    
538	T T T T T T T T T T T T T T T T T T T T T    
539	G G G G G G G G G G G G G G G G G G G G G    
540	T T T T T T T T T T T T T T T T T T T T T    
541	T T T T T T T T T T T T T T T T T T T T T    
542	C C C C C C C C C C C C C C C C C C C C C    
543	A A A A A A A A A A A A A A A A A A A A A    
544	A A A A A A A A A A A A A A A A A A A A A    
545	T T T T T T T T T T T T T T T T T T T T T    
546	G G G G G G G G G G G G G G G G G G G G G    
547	A A A A A A A A A A A A A A A A A A A A A    
548	C C C C C C C C C C C C C C C C C C C C C    
549	C C C C C C C C C C C C C C C C C C C C C    
550	A A A A A A A A A A A A A A A A A A A A A    
551	C C C C C C C C C C C C C C C C C C C C C    
552	C C C C C C C C C C C C C C C C C C C C C    
553	C C C C C C C C C C C C C C C C C C C C C    
554	T T T T T T T T T T T T T T T T T T T T T    
555	C C C C C C C C C C C C C C C C C C C C C    
556	C C C C C C C C C C C C C C C C C C C C C    
557	G G G G G G G G G G G G G G G G G G G G G    
558	G G G G G G G G G G G G G G G G G G G G G    
559	C C C C C C C C C C C C C C C C C C C C C    
560	C C C C C C C C C C C C C C C C C C C C C    
561	T T T T T T T T T T T T T T T T T T T T T    
562	T T T T T T T T T T T T T T T T T T T T T    
563	C C C C C C C C C C C C C C C C C C C C C    
564	T T T T T T T T T T T T T T T T T T T T T    
565	G G G G G G G G G G G G G G G G G G G G G    
566	T T T T T T T T T T T T T T T T T T T T T    
567	G G G G G G G G G G G G G G G G G G G G G    
568	A A A A A A A A A A A A A A A A A A A A A    
569	C C C C C C C C C C C C C C C C C C C C C    
570	A A A A A A A A A A A A A A A A A A A A A    
571	A A A A A A A A A A A A A A A A A A A A A    
572	T T T T T T T T T T T T T T T T T T T T T    
573	C C C C C C C C C C C C C C C C C C C C C    
574	T T T T T T T T T T T T T T T T T T T T T    
575	C C C C C C C C C C C C C C C C C C C C C    
576	C C C C C C C C C C C C C C C C C C C C C    
577	G G G G G G G G G G G G G G G G G G G G G    
578	G G G G G G G G G G G G G G G G G G G G G    
579	C C C C C C C C C C C C C C C C C C C C C    
580	T T T T T T T T T T T T T T T T T T T T T    
581	G G G G G G G G G G G G G G G G G G G G G    
582	A A A A A A A A A A A A A A A A A A A A A    
583	T T T T T T T T T T T T T T T T T T T T T    
584	. C C C C C C C C C C C C C C C C C C C C    
585	. G G G G G G G G G G G G G G G G G G G G    
586	. T T T T T T T T T T T T T T T T T T T T    
587	. C C C C C C C C C C C C C C C C C C C C    
588	. C C C C C C C C C C C C C C C C C C C C    
589	. C T T T T T T T T T T T T T T T T T T T    
590	. . C C C C C C C C C C C C C C C C C C C    
591	. . G G G G G G G G G G G G G G G G G G G    
592	. . C C C C C C C C C C C C C C C C C C C    
593	. . C C C C C C C C C C C C C C C C C C C    
594	. . T T T T T T T T T T T T T T T T T T T    
595	. . T T T T T T T T T T T T T T T T T T T    
596	. . T T T T T T T T T T T T T T T T T T T    
597	. . A A A A A A A A A A A A A A A A A A A    
598	. . C C C C C C C C C C C C C C C C C C C    
599	. . C C C C C C C C C C C C C C C C C C C    
600	. . C C C C C C C C C C C C C C C C C C C    
601	. . C C C C C C C C C C C C C C C C C C C    
602	. . T T T T T T T T T T T T T T T T T T T    
603	. . C C C C C C C C C C C C C C C C C C C    
604	. . T T T T T T T T T T T T T T T T T T T    
605	. . T T T T T T T T T T T T T T T T T T T    
606	. . C C C C C C C C C C C C C C C C C C C    
607	. . T T T T T T T T T T T T T T T T T T T    
608	. . C C C C C C C C C C C C C C C C C C C    
609	. . C C C C C C C C C C C C C C C C C C C    
610	. . T - - - - - - T T T T T T T T T T - -  8	 indel  
611	. . C - - - - - - C C C C C C C C C C - -  8	 indel  
612	. . C - - - - - - C C C C C C C C C C - -  8	 indel  
613	. . A A A A A A A A A A A A A A A A A A A    
614	. . C C C C C C C C C C C C C C C C C C C    
615	. . . G G G G G G G G G G G G G G G G G G    
616	. . . G G G G G G G G G G G G G G G G G G    
617	. . . A A A A A A A A A A A A A A A A A A    
618	. . . C C C C C C C C C C C C C C C C C C    
619	. . . T T T T T T T T T T T T T T T T T T    
620	. . . C C C C C C C C C C C C C C C C C C    
621	. . . C C C C C C C C C C C C C C C C C C    
622	. . . T T T T T T T T T T T T T T T T T T    
623	. . . C C C C C C C C C C C C C C C C C C    
624	. . . C C C C C C C C C C C C C C C C C C    
625	. . . T T T T T T T T T T T T T T T T T T    
626	. . . T T T T T T T T T T T T T T T T T T    
627	. . . G G G G G G G G G G G G G G G G G G    
628	. . . A A A A A A A A A A A A A A A A A A    
629	. . . C C C C C C C C C C C C C C C C C C    
630	. . . G G G G G G G G G G G G G G G G G G    
631	. . . C C C C C C C C C C C C C C C C C C    
632	. . . T T T T T T T T T T T T T T T T T T    
633	. . . C C C C C C C C C C C C C C C C C C    
634	. . . G G G G G G G G G G G G G G G G G G    
635	. . . C C C C C C C C C C C C C C C C C C    
636	. . . C C C C C C C C C C C C C C C C C C    
637	. . . T T T T T T T T T T T T T T T T T T    
638	. . . T T T T T T T T T T T T T T T T T T    
639	. . . G G G G G G G G G G G G G G G G G G    
640	. . . C C C C C C C C C C C C C C C C C C    
641	. . . G G G G G G G G G G G G G G G G G G    
642	. . . C C C C C C C C C C C C C C C C C C    
643	. . . T T T T T T T T T T T T T T T T T T    
644	. . . T T T T T T T T T T T T T T T T T T    
645	. . . C C C C C C C C C C C C C C C C C C    
646	. . . A A A A A A A A A A A A A A A A A A    
647	. . . A A A A A A A A A A A A A A A A A A    
648	. . . A A A A A A A A A A A A A A A A A A    
649	. . . A A A A A A A A A A A A A A A A A A    
650	. . . C C C C C C C C C C C C C C C C C C    
651	. . . A A A A A A A A A A A A A A A A A A    
652	. . . C C C C C C C C C C C C C C C C C C    
653	. . . C C C C G G G G G G G G G G G G G G    
654	. . . . . . . G G A A A A A A A A A A A A    
655	. . . . . . . . . C C C C C C C C C C C C    
656	. . . . . . . . . C C C C C C C C C C C C    
657	. . . . . . . . . T T T T T T T T T T T T    
658	. . . . . . . . . G G G G G G G G G G G G    
659	. . . . . . . . . C C C C C C C C C C C C    
660	. . . . . . . . . T T T T T T T T T T T T    
661	. . . . . . . . . T T T T T T T T T T T T    
662	. . . . . . . . . T T T T T T T T T T T T    
663	. . . . . . . . . A . . A A A A A A A A A    
664	. . . . . . . . . . . . T T . T T T T T T    
665	. . . . . . . . . . . . T T . T T T T T T    
666	. . . . . . . . . . . . A A . A A A A A A    
667	. . . . . . . . . . . . C C . C C C C C C    
668	. . . . . . . . . . . . G G . G G G G G G    
669	. . . . . . . . . . . . G G . G G G G G G    
670	. . . . . . . . . . . . G G . G G G G G G    
671	. . . . . . . . . . . . A A . A A A A A A    
672	. . . . . . . . . . . . C C . C C C C C C    
673	. . . . . . . . . . . . G G . G G G G G G    
674	. . . . . . . . . . . . C C . C C C C C C    
675	. . . . . . . . . . . . C C . C C C C C C    
676	. . . . . . . . . . . . A A . A A A A A A    
677	. . . . . . . . . . . . T T . T T T T T T    
678	. . . . . . . . . . . . T T . T T T T T T    
679	. . . . . . . . . . . . G G . G G G G G G    
680	. . . . . . . . . . . . C C . C C C C C C    
681	. . . . . . . . . . . . T T . T T T T T T    
682	. . . . . . . . . . . . . G . G G G G G G    
683	. . . . . . . . . . . . . T . T T T T T T    
684	. . . . . . . . . . . . . . . T T T T T T    
685	. . . . . . . . . . . . . . . . . . . A A    
686	. . . . . . . . . . . . . . . . . . . T T    
687	. . . . . . . . . . . . . . . . . . . T T    
688	. . . . . . . . . . . . . . . . . . . G G    
689	. . . . . . . . . . . . . . . . . . . G G    
690	. . . . . . . . . . . . . . . . . . . T T    
691	. . . . . . . . . . . . . . . . . . . C C    
692	. . . . . . . . . . . . . . . . . . . T T    
693	. . . . . . . . . . . . . . . . . . . T T    
694	. . . . . . . . . . . . . . . . . . . G G    
695	. . . . . . . . . . . . . . . . . . . T T    
696	. . . . . . . . . . . . . . . . . . . G G    
697	. . . . . . . . . . . . . . . . . . . T T    
698	. . . . . . . . . . . . . . . . . . . A A    
699	. . . . . . . . . . . . . . . . . . . A A    
700	. . . . . . . . . . . . . . . . . . . T T    
701	. . . . . . . . . . . . . . . . . . . G G    
702	. . . . . . . . . . . . . . . . . . . A A    
703	. . . . . . . . . . . . . . . . . . . G G    
704	. . . . . . . . . . . . . . . . . . . T T    
705	. . . . . . . . . . . . . . . . . . . G G    
706	. . . . . . . . . . . . . . . . . . . A A    
707	. . . . . . . . . . . . . . . . . . . A A    
708	. . . . . . . . . . . . . . . . . . . C C    
709	. . . . . . . . . . . . . . . . . . . T T    
710	. . . . . . . . . . . . . . . . . . . T T    
711	. . . . . . . . . . . . . . . . . . . T T    
712	. . . . . . . . . . . . . . . . . . . C C    
713	. . . . . . . . . . . . . . . . . . . G G    
714	. . . . . . . . . . . . . . . . . . . C C    
715	. . . . . . . . . . . . . . . . . . . G G    
716	. . . . . . . . . . . . . . . . . . . C C    
717	. . . . . . . . . . . . . . . . . . . G G    
718	. . . . . . . . . . . . . . . . . . . T T    
719	. . . . . . . . . . . . . . . . . . . C C    
720	. . . . . . . . . . . . . . . . . . . C C    
721	. . . . . . . . . . . . . . . . . . . C C    
722	. . . . . . . . . . . . . . . . . . . C C    
723	. . . . . . . . . . . . . . . . . . . C C
```
